# Functional Brain Dysfunction in Patients with Benign Childhood Epilepsy as Revealed by Graph Theory

**DOI:** 10.1371/journal.pone.0139228

**Published:** 2015-10-02

**Authors:** Azeez Adebimpe, Ardalan Aarabi, Emilie Bourel-Ponchel, Mahdi Mahmoudzadeh, Fabrice Wallois

**Affiliations:** 1 Institut National de la Santé et de la Recherche Médicale (INSERM U1105), Centre Universitaire de Recheche en Santé (CURS), University Hospital, Amiens, France; 2 Explorations Fonctionnelles du Système Nerveux (EFSN) pédiatrique, University Hospital, Amiens, France; University of Minnesota, UNITED STATES

## Abstract

There is growing evidence that brain networks are altered in epileptic subjects. In this study, we investigated the functional connectivity and brain network properties of benign childhood epilepsy with centrotemporal spikes using graph theory. Benign childhood epilepsy with centrotemporal spikes is the most common form of idiopathic epilepsy in young children under the age of 16 years. High-density EEG data were recorded from patients and controls in resting state with eyes closed. Data were preprocessed and spike and spike-free segments were selected for analysis. Phase locking value was calculated for all paired combinations of channels and for five frequency bands (δ, θ, α, β_1_ and β_2_). We computed the degree and small-world parameters—clustering coefficient (C) and path length (L)—and compared the two patient conditions to controls. A higher degree at epileptic zones during interictal epileptic spikes (IES) was observed in all frequency bands. Both patient conditions reduced connection at the occipital and right frontal regions close to the epileptic zone in the α band. The “small-world” features (high C and short L) were deviated in patients compared to controls. A changed from an ordered network in the δ band to a more randomly organized network in the α band was observed in patients compared to healthy controls. These findings show that the benign epileptic brain network is disrupted not only at the epileptic zone, but also in other brain regions especially frontal regions.

## Introduction

Benign childhood epilepsy with centrotemporal spikes (BCECTS) is the most common childhood epilepsy syndrome, usually affecting the children under the age of 16 years [1,2]. Several studies have demonstrated different cognitive impairments [3] including frontal dysfunction [4] in patients with benign epilepsy with no evidence of large structural changes compared to other forms of epilepsy [5,6]. However, all forms of epilepsy are associated with abnormal brain activity and impaired neural processing as a result of unstable brain dynamics and networks [7]. Dynamic changes of epileptic brain networks are believed to be caused by dysregulation of neurotransmitters leading to abnormal electrical activity [8].

Abnormal synchronization of neurons, probably due to changes in the spatial organization of the neural networks, is also thought to contribute to the generation and propagation of epileptic seizures [9]. Graph theory is a promising mathematical approach to study topological characteristics of both local and long distance brain functional connectivity using fMRI, EEG and MEG [10–12]. Graph analysis of brain connectivity has revealed reconfiguration of both structural and functional connections between different neural networks in several brain disorders [13]. Few studies have compared the resting state of healthy controls to that of epileptic patients at network level [14]. The characterization of the dynamics of the cortical networks in scalp EEG and electrocorticography (ECoG) in epileptic patients during the resting state has demonstrated disruptions in global and regional brain networks [15–17].

A large number of graph metrics have been proposed, two of which, clustering coefficient and path length [18], have been mostly used to characterize the functional connectivity of human brain networks [13,19]. The clustering coefficient (C) is a measure of functional segregation quantifying the presence of locally connected groups known as clusters or modules, which indicate segregated neural processing [18]. Path length (L), however, measures network integration by estimating the effective communications between different brain regions [18]. A graph comprising numerous local and few long-distance connections (high C and short L) most closely corresponds to the optimal network, called the ‘small-world network (SWN)’, which is intermediate between ordered (high C and long L) and random networks (low C and short L) [20,21]. However, various types of neurological diseases, including epilepsy, have been reported to deviate from SWN properties [22–24].

In this study, we investigated changes in brain functional connectivity in BCECTS patients compared to healthy controls in various frequency bands using high-density resting EEG data under the eyes-closed condition. For this purpose, we quantified the topological properties of the BCECTS brain networks at rest during periods with and without interictal epileptic spikes (IES) by estimating the clustering coefficient (C) and path length (L) from the functional connectivity matrices reconstructed with phase locking value (PLV). We also estimated the degree of centrality that measures the number of connections between a particular node (a specific brain region) to other nodes [18]. In summary, this study was designed to investigate whether BCECTS functional brain networks in the presence or absence of interictal spikes exhibited characteristic changes in small-world network features.

## Materials and Methods

### Subjects

Our study was conducted at Amiens University Hospital (Amiens, France) and approved by the hospital’s ethics committee (CPP Nord-Ouest 2, approval No. 2011-A00782-39). Written consent approved by the ethics committee was obtained from parents/caregivers. Eight healthy adolescents (9 ± 0.21 years old) and nine young patients (9 ± 0.24 years old) with BCECTS were included in this study. All patients presented right centrotemporal spikes (see S1 Fig) and were free of any other neurological disorder at the time of the study.

### EEG recording and preprocessing

On average, thirteen-minute high-resolution EEG data were recorded from each subject resting comfortably in a supine position in a quiet room. EEG data were recorded with 64 channels based on the international 10–10 system and a sampling rate of 256 Hz. An average reference montage was used for all of the analysis. Data were filtered offline between 0.5 to 30 Hz to exclude high-frequency noise including muscle activities. To identify EEG portions with ocular and movement artifacts, which were excluded from the analysis, the EEG recordings were first normalized by the Z-score transformation and then processed semi-automatically (with visual inspection) using a simple threshold method (threshold set to the mean of the z-score distribution for each channel) as it was implemented in Fieldtrip software (http://www.fieldtriptoolbox.org/tutorial/visual_artifact_rejection) [25]. Two neurophysiologists visually inspected the filtered data in order to identify spikes segment and artifacts.

Artifact-free portions of the EEG data were partitioned into two-second non-overlapping segments. Five segments were randomly selected for each of the control subjects (CON). Two conditions were defined for the epileptic group: 5 segments with interictal spikes (With Spike Condition—WSC) and 5 spike-free segments (No Spike Condition—NSC), all randomly selected. On average, the WSC EEG segments contained 7 spikes considered as a requirement to ensure homogeneity across the patients.

### Functional Connectivity

Pairwise correlations between all EEG channels were computed with the Phase Locking Value (PLV) [26,27]. Briefly, the PLV belongs to the family of phase synchronization values that are used to estimate functional connectivity between two signals based on their relative phase differences. To calculate the PLV, we first filtered the data into frequency bands (δ (0.5–3.5 Hz), θ (4–8 Hz), α (8.5–13 Hz), β_1_ (13.5–20 Hz) and β_2_ (20.5–30 Hz) using zero-phase forward and reverse digital filtering. The analytical signals were obtained by Hilbert transformation of the filtered signals. The Hilbert transformed signals consisted of the instantaneous amplitude and phase of the signals. The phase angle (φ) was used to compute the PLV. The PLV ranged from 0 to 1, with 0 and 1 indicating no connection and maximum connection between any given pair of signals, respectively. The end-result of computing the PLV for all paired combinations of channels was a square matrix of size 63 (number of EEG channels), in which each entry N_x,y_ (= N_y,x_) contained the PLV for channels x and y (see Supporting information for more details).

### Computation of graph theory parameters

A graph is a basic topographical representation of a network consisting of nodes or vertices (in this case brain regions or electrodes) and edges (correlation between nodes). In this study, the network consisted of 63 vertices (electrodes) connected by edge weights (or elements) between all pairs of channels. The first step in applying graph theoretical analysis to functional connectivity matrices consists of converting the matrix into a binary graph, in which the edges either exist (1) or do not exist (0), i.e, with no graded values. Functional connectivity matrices were converted to binary graphs by applying an optimal threshold, τ above/below which connectivity values were set to 1/0. This operation transformed functional connectivity matrices to binary adjacency matrices, which was then followed by computation of graph metrics.

For each subject and frequency band, we determined the optimal threshold using an iterative method to make sure that the proportion and global spatial distribution of connections between brain regions were similar across subjects. We did not, however, choose a single threshold for all frequency bands mainly because it could lead to false positives in some frequency bands resulted from highly disconnected or over-densely networks [28]. Our threshold optimization procedure was based on the computation of the degree, which is defined for each node as the number of links connected to the node. The degree is used to measure the importance of individual nodes (nodes with a high degree of interaction with other nodes). The optimal thresholds were iteratively determined by means of the following procedure. First, for each functional connectivity matrix, we set the threshold to one standard deviation above the median connectivity value. We then calculated the mean degree for the whole brain network. The optimal threshold was determined under two conditions, (i) the mean degree must not be less than 2ln(N), and (ii) at least 95% of nodes must be connected to one or more nodes [28,29] (see SI for more information). These conditions were then used to optimize the connection strength, which was used to increase the signal-to-noise ratio and to reduce false-positive edges in the graph [29].

To investigate the global topology of large-scale brain functional networks in patients and controls, the optimal threshold for each subject and frequency band was applied to the functional connectivity matrix for computation of degree of the whole brain network. We then investigated whether the occurrence of interictal spikes could change the small-world network features (C and L) in BCECTS brain networks. The clustering coefficient of a node is the ratio of the number of actual edges to the total number of potential edges adjacent to the node. The clustering coefficient was computed for all nodes and averaged (mean clustering coefficient, C). The path length is the mean shortest path connecting any two nodes of the graph and indicates how well the nodes are interconnected or integrated [18,20]. Similarly, we computed the mean path length (L) of the whole brain network (see SI for mathematical description). C and L were computed as a function of network density defined as the ratio of the actual number of edges in the graph to the total number of possible edges. In order to detect significant differences in network organization between the groups and to minimize the number of false (or noisy) edges in the networks, we only investigated strong connections by changing the network density from 30% to 60% in steps of 5% based on previous studies [30,31].

### Statistical analysis

Nonparametric permutation testing was used for all graph parameters with correction for multiple comparisons including *post hoc* tests with a p-value ≤ 0.05. A total of 1,000 permutations were used to determine the significance level for each test [32]

All computations and statistical analyses were performed in Matlab with custom scripts and open source toolboxes: Fieldtrip (http://fieldtrip.fcdonders.nl/), EEGLAB (for 3D topological plots, http://sccn.ucsd.edu/eeglab/), and the brain connectivity toolbox (for graph parameter computations, https://sites.google.com/site/bctnet/).

## Results

### Synchronization and degree

**Fig 1:**
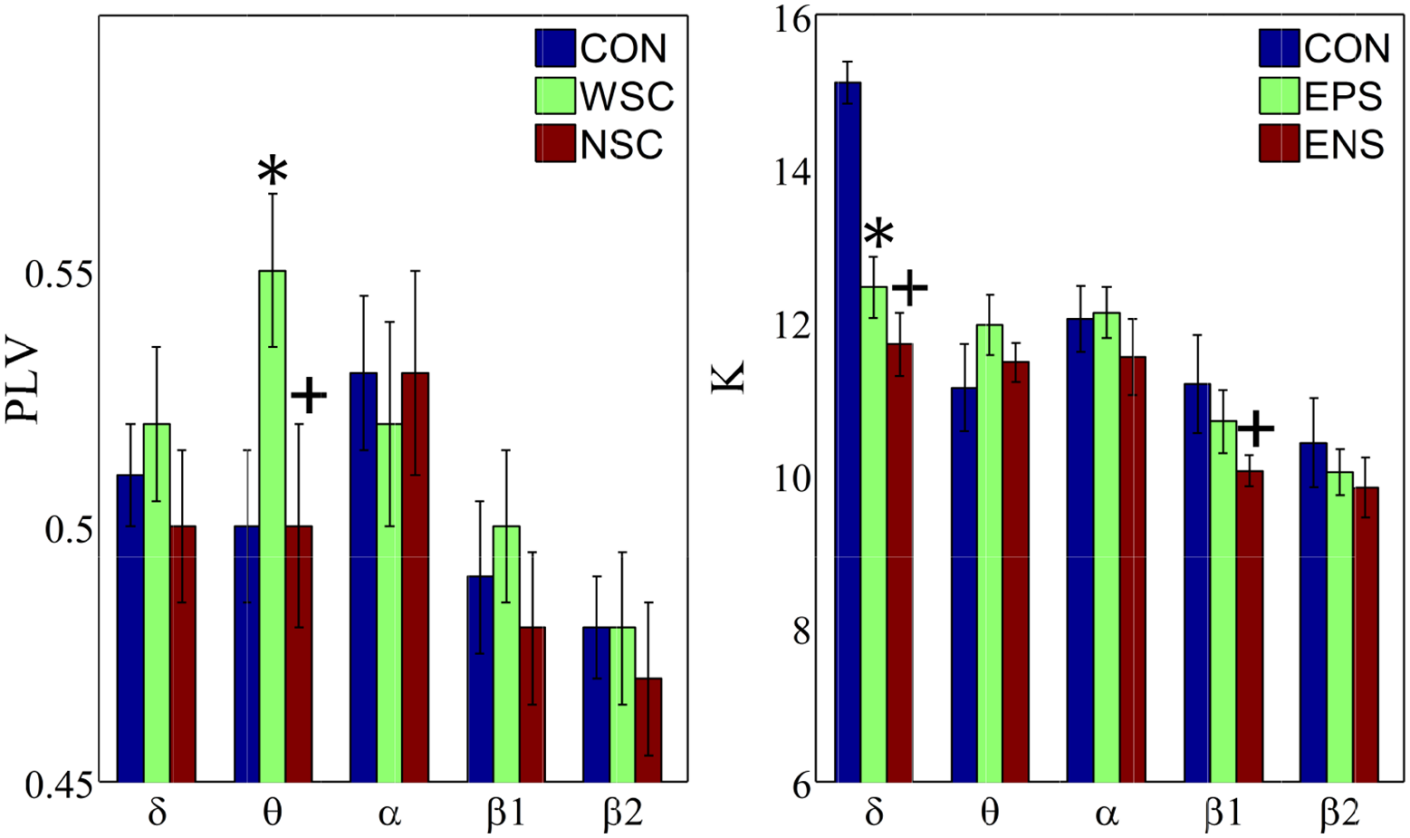
Group mean Phase Locking Value (PLV) and group mean degree (K). Error bars show standard errors with 95% confidence intervals. CON, WSC and NSC indicate the control, with spike and no spike conditions, respectively. Statistical significance is denoted by * (WSC vs CON) and + (NSC vs CON) with p<0.05.

The mean PLV was analysed under the three conditions in order to investigate differences in synchronization between patients and controls (Fig 1). As shown, the mean PLV under the with-spike condition exhibited significantly higher values in the θ band compared to the other two conditions (CON and NSC). No significant differences in synchronization were observed between the groups in the other frequency bands.

Compared to controls, BCECTS patients were characterized by significantly lower mean degree values in the δ band (under both WSC and NSC) and in the β_1_ band (only under NSC).

We also investigated whether our results were affected by the precise choice of the thresholds. As shown in Table 1, within each group the variations in PLV and K due to changes in the optimal thresholds were very small for each particular frequency band.

**Table 1:** Mean and range of changes (at 95% confidence interval) of phase locking value (PLV), threshold (*τ*) and degree (K) computed for each group and frequency band.

	Frequency band	PLV	τ	K
**CON**	δ	0.51±0.02	0.59±0.01	15.12±1.40
	θ	0.50±0.03	0.60±0.01	11.14±1.13
	α	0.53±0.03	0.63±0.02	12.04±0.86
	β_1_	0.49±0.03	0.57±0.01	11.19±1.28
	β_2_	0.48±0.02	0.54±0.01	10.42±1.16
**WSC**	δ	0.52±0.03	0.63±0.02	12.45±0.80
	θ	0.55±0.03	0.64±0.01	11.96±0.79
	α	0.52±0.04	0.62±0.03	12.12±0.66
	β_1_	0.50±0.03	0.58±0.01	10.70±0.81
	β_2_	0.48±0.03	0.55±0.00	10.04±0.59
**NSC**	δ	0.50±0.03	0.60±0.03	11.71±0.82
	θ	0.50±0.04	0.60±0.02	11.47±0.50
	α	0.53±0.04	0.62±0.04	11.54±1.00
	β_1_	0.48±0.03	0.55±0.01	10.06±0.41
	β_2_	0.47±0.03	0.53±0.01	9.83±0.79

**Fig 2:**
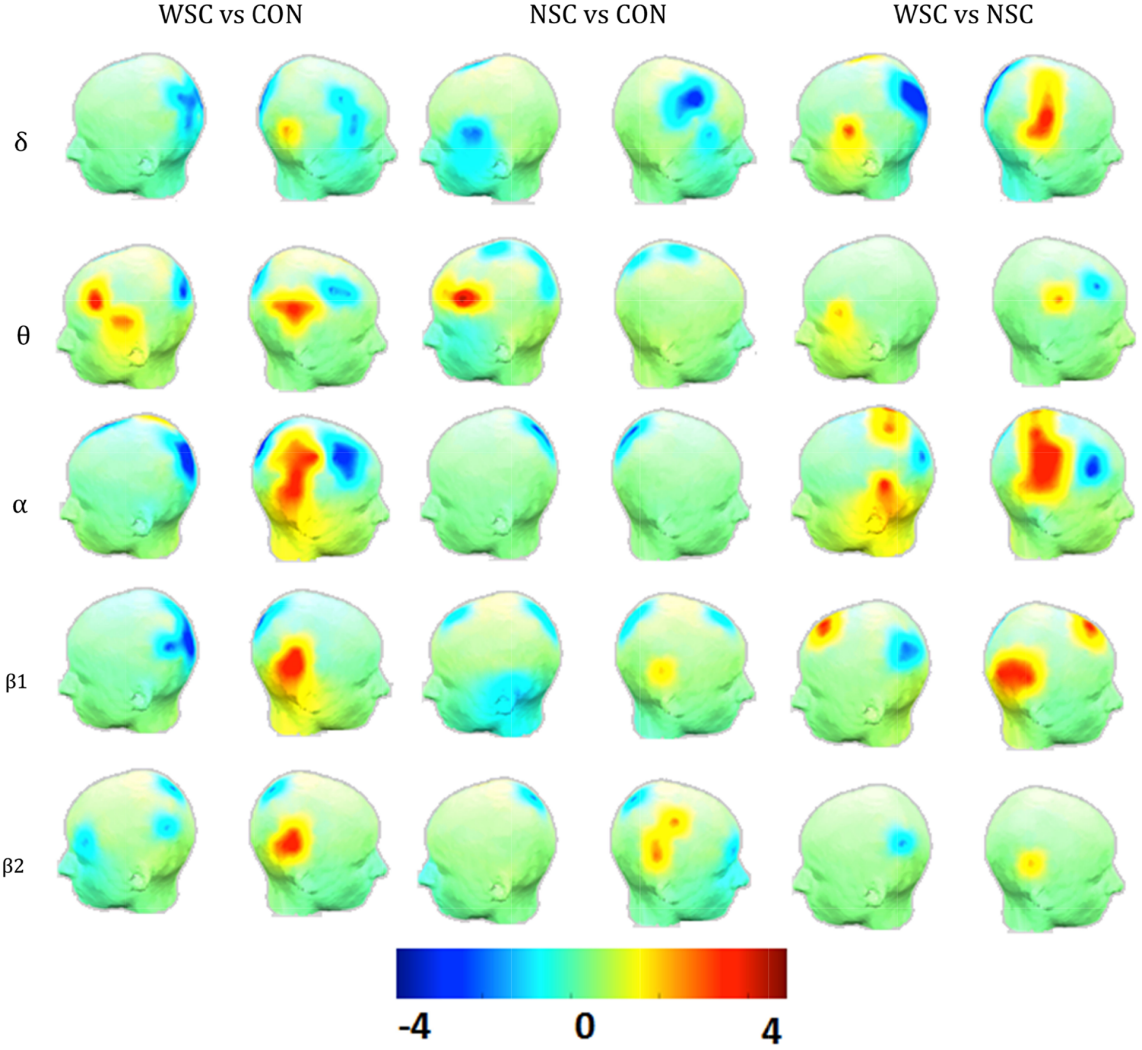
Statistical difference (t-value) maps of degree between the control (CON) and the epileptic groups (WSC and NSC). The color bar indicates the t values projected onto a standardized head shape. The significant increase (indicated by red) and decrease (indicated by blue) in degree have been represented, respectively, by positive and negative t values resulted from statistical comparisons between (WSC and CON), (NSC and CON), and (WSC and NSC).

We further investigated the regional differences in the degree (K) distribution between the groups (Fig 2). In the presence of IES (WSC vs. CON), K significantly increased in the right centrotemporal region (IES generator region), and decreased in the occipital region in almost all frequency bands. Right frontocentral areas exhibited lower K values in mid-range frequencies (θ and α). On the contralateral side, however, the degree significantly increased in the left frontal and frontotemporal regions in the θ band.

In the absence of IES (NSC vs. CON), K increased in the left frontal region in the θ band and in right centrotemporal areas in high frequencies (β_1_ and β_2_). In the mid- and high-range frequencies, patients exhibited relatively lower values of degree in occipital areas.

Compared to NSC, WSC was characterized with significant increases of K in the right centrotemporal regions in the δ and α band, and in the right parietotemporal regions in the β_1_ band. The degree decreased significantly in occipital areas in all frequencies except in the θ band, in the right frontocentral region close to the spike generation zone in mid-range frequencies (θ and α), and in the left posterior region in the β_1_ band.

### Clustering coefficient and path length

**Fig 3:**
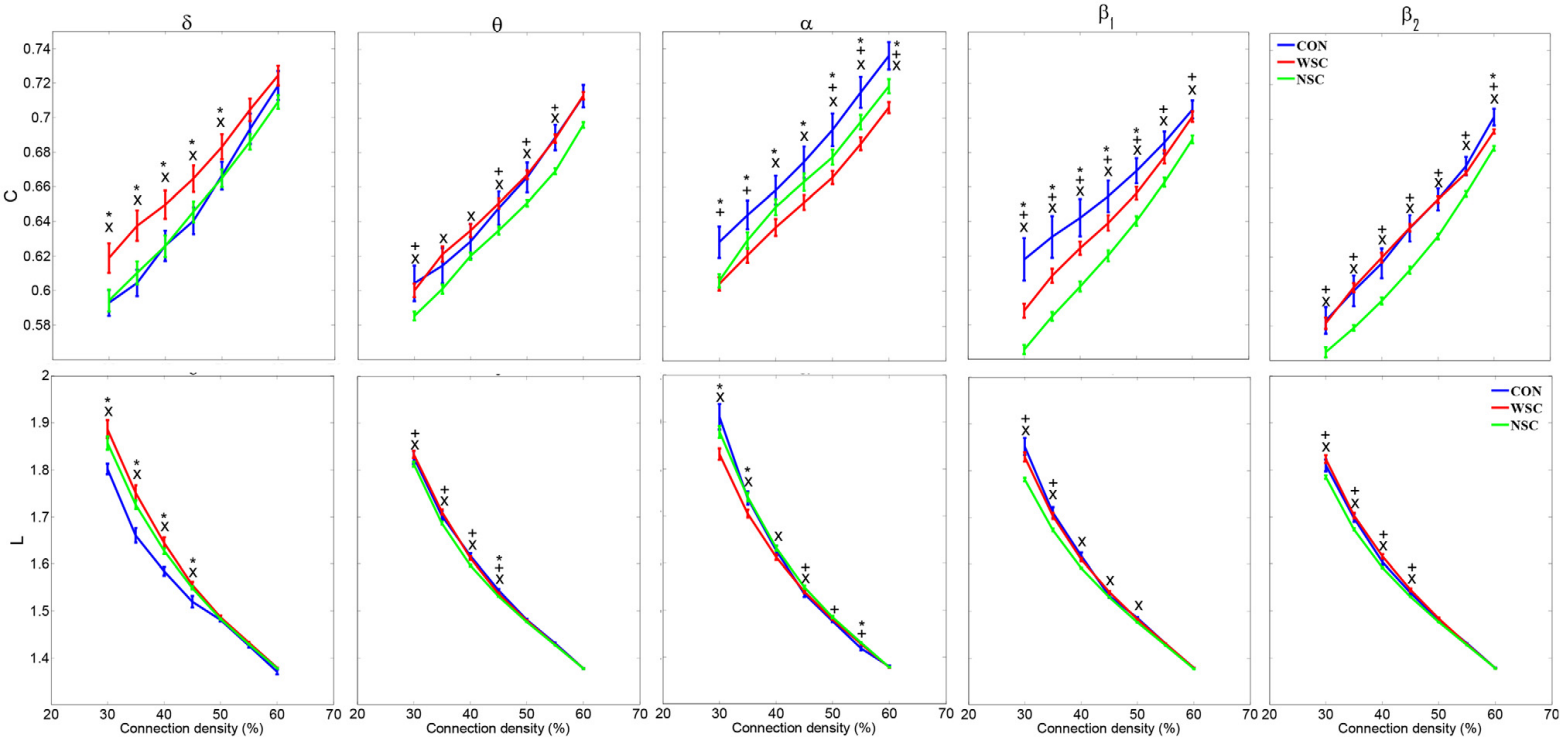
Mean clustering coefficient (C) and mean path length (L) as a function of network density for each frequency band. The error bars represent standard error with 95% confidence intervals. CON, WSC and NSC indicate the control, with spike and no spike conditions, respectively. Statistical significance is denoted by * (WSC vs. CON), + (NSC vs. CON) and × (WSC vs. NSC) with p<0.05.

Fig 3 shows the mean clustering coefficient (C) and mean path length (L) computed for each condition.

In low frequencies (δ), the presence of IES (WSC) in the EEG segments significantly increased C at low connection densities (<50%) in comparison to the other two conditions. Compared to CON, both epileptic conditions exhibited shorter path length at connection densities up to 45%.

In the θ band, compared to NSC, WSC and CON showed significantly higher C (for all connection densities) and longer L (for connection densities less than 40%). No significant differences in clustering coefficients were observed between WSC and CON at almost all connection densities.

In the α band, both NSC and WSC compared to CON, and WSC compared to NSC exhibited significantly lower C values for almost all connection densities. Similar differences in L were found between the groups on a less significant level. The only exception was shorter path lengths found for WSC compared to CON and NSC at connection densities up to 45%.

In higher frequencies (β_1_ and β_2_), lower C (over all connection densities) and shorter L (for connection densities less than 45%) were found under NSC (vs. WSC and CON). In the β_1_ band, compared to CON, WSC was characterized by significantly lower clustering coefficients. Table 2 roughly summarizes overall significant differences between the conditions.

**Table 2:** Summary of differences in mean clustering coefficient (C) and mean path length (L) between the three conditions as shown in Fig 3.

	C					L				
	δ	θ	α	β_1_	β_2_	δ	θ	α	β_1_	β_2_
**WSC vs. NSC**	↑	↑	↓	↑	↑	NS	↑	↓	↑	↑
**WSC vs. CON**	↑	NS	↓	↓	NS	↑	NS	↓	NS	NS
**NSC vs. CON**	NS	↓	↓	↓	↓	↑	↓	↓	↓	↓

↑ and ↓ indicate significant increase and decrease in C and L between conditions, respectively. NS represents non-significant differences.

## Discussion

This study investigated differences in the brain functional connectivity between BCECTS patients and healthy controls. Statistical dependencies between EEG time-series recorded from different brain regions were investigated by computing functional interactions using PLV as a measure of phase synchronization as well as graph metrics. The brain functional connectivity of BCECTS patients was found to be disrupted in terms of synchronization and degree of connectivity not only in the IES zone but also in frontal and temporal areas. Deviations from small-world features and network parameters were also observed in various frequency bands in BCECTS patients regardless of the presence or absence of spikes in EEG segments.

Many studies have shown that neurological diseases in children [33,34] are associated with differences in the level of synchronization compared to healthy controls. Compared to the other frequency bands, we found higher synchronization values in the α band in healthy subjects (Fig 1). The increased α synchronization is generally accepted to be due to the increased alpha activation under the eyes-closed resting state of the brain [35]. However, compared to controls, BCECTS patients exhibited significantly increased θ and decreased α synchronization under the with-spike condition. Our observations are consistent with those reported in patients with temporal lobe epilepsy [30], who presented significantly increased synchronization in the θ band, but non-significantly decreased synchronization in the α band. The increase of θ synchronization is commonly observed, not only in epilepsy [36,37] but also in other neurological diseases such as Parkinson’s disease [38] and Alzheimer’s disease [39].

### Interictal spikes disrupt the global topology of brain functional connectivity

The disruption of brain dynamics in BCECTS patients probably results in higher levels of synchronization in some regions of the brain (especially in the epileptogenic zone) and lower levels of synchronization in other regions due to epileptic spikes. Although there is little evidence to suggest that BCECTS is associated with structural brain abnormalities [40], we found strong frequency-dependent changes in the degree as a measure of centrality or information coordination implying disrupted functional connectivity in the epileptic zone in BCECTS patients.

However, increased and decreased degrees in other regions, notably frontal and occipital, support the idea that disruption of brain functional connectivity in BCECTS patients is unlikely to be restricted to the epileptogenic zone. This finding may reflect the functional reorganization of the BCECTS brain network topology.

### Functional disruption of BCECTS frontal networks

Our results revealed functional dysfunction of frontal brain networks in the presence/absence of interictal epileptic spikes. Compared to controls, BCECTS patients were characterized by a reduced degree in the right frontal region in the α band and an increased degree in the left frontal region in the θ band. These observations suggest functional network reorganization in the frontal regions regardless of the presence or absence of spikes in EEG segments. This frontal functional dysfunction may confirm the results of longitudinal MRI studies [41], which suggested that learning and memory difficulties in BCECTS patients may be associated with serial changes in the frontal and prefrontal lobes [42–44].

The alteration of brain functional connectivity in the absence of IES does not exclusively affect the frontal regions; it also involves the occipital region. This spatial pattern of changes was also observed in the presence of IES, especially in the α band. The decreased degree in the α band in the posterior region under the epileptic conditions may support the disruption of functional brain connectivity in BCECTS patients even though the differences in global synchronization did not reach statistical significance. Since in healthy controls the functional connectivity has been shown to increase in frontal and posterior regions under the eyes-closed condition in the α band [45], in BCECTS patients the reduced α degree at the occipital region may support their poor visual spatial memory [46].

### Deviation from small-world network features

There has been a growing interest in small-world analysis of brain networks in various neurological diseases [13]. The small-world network features of healthy controls have been compared to different brain diseases, such as schizophrenia [47], depression [48], Alzheimer’s disease [24] and various types of epilepsy [14]. Most of these neurological diseases are associated with lower clustering coefficients and shorter path lengths compared to healthy controls.

The present study demonstrated frequency-dependent alterations of small-world features and network parameters in BCECTS patients in the presence and in the absence of IES. In the δ band, patients under the WSC condition exhibited higher C and long L compared to controls, implying that, at very low frequencies, BCECTS brain networks exhibit more functionally ordered organization in the presence of IES. This observation is consistent with the findings observed in other types of epilepsy [49]. In higher frequency bands (α and β_1_), patients showed lower C values under both WSC and NSC compared to controls. The simultaneous decrease in C and L in the β_1_ band may indicate loss of global processing and stronger integration between long-distance brain regions, which can be interpreted as increased functional interaction between long-range brain connections in BCECTS patients.

Interestingly, a switch in the brain network functional organization in the θ and α bands was observed when comparing WSC and NSC conditions. BCECTS patients’ brain networks tended to change from a more randomly organized network (low C, short L) in the α band to a functionally ordered network (high C, long L) in the θ and β bands due to IES. The global increase in C in the presence of IES (excluding the α band) reflects orderly connection of IES brain networks. This finding is in agreement with studies on the other types of epilepsies using intracranial EEG and ECoG [16,50]. Small-world networks allow more rapid information processing and learning than either random or regular networks [51] and the results may suggest that the cognitive impairments observed in BCECTS may be associated with rapid changes in the functional reconfiguration of BCECTS brain networks. Although the shorter path length in higher frequency bands (in the α band for WSC and the β band for NSC has been shown to support effective interactions between and across brain regions [21], BCECTS brain networks may more closely resemble randomly organized networks.

In the absence of IES, C was lower in all frequency bands and L was lower in the θ and β bands in BCECTS patients compared to controls, indicating that, in the absence of IES, the BCECTS brain network is less ordered regardless of the frequency band except for the α band. It is generally believed that random networks ensure even better synchronization than small-world networks [52], as pathological random networks present rapid phase transition that could lead to the onset of interictal epileptic discharges. In agreement with our observations, temporal lobe epilepsy [30] has been characterized by lower C and shorter L in the α band compared to controls. These discrepancies between TLE and BCECTS are clinically relevant and may constitute a specific biomarker of the type of epilepsy. However, to confirm our findings, further study with a larger population of patients will be needed.

### Limitations and future directions

A potential limitation of our study is the use of scalp EEG for the functional connectivity analysis. In studies on epilepsy, scalp EEG is usually used for EEG source imaging and/or functional connectivity analysis [53], are employed for localizing the epileptogenic foci and investigating the functional organization of the epileptic cortical networks, respectively. In our study, we used PLV as a measure of undirected functional connectivity between electrodes to explore the global topology and dynamics of the interactions between large-scale brain regions during the interictal state over a range of frequencies. The functional connectivity analysis in the sensor space might provide inaccurate information on the overall organization of the cortical region mainly because EEG electrodes detect spatially averaged overlapping EEG signals from several brain sources or the signal generated by a focal cerebral source can be detected by nearby electrodes [54].

Our main direction of future work will be to use the EEG source space connectivity tools such as the directed transfer function (DTF)[55] or the Phase-Slope Index (PSI) [56] for the identification and characterization of the cortical networks involved in the interictal states.

In patients with epilepsy, the functional connectivity analysis using DTF has provided promising results using scalp EEG [57] and ECoG [58] for exploring the directed functional connectivity between cortical regions and the propagation of activation [58,59]. In general, the EEG functional connectivity analysis between neighbouring voxels might lead to spurious and over-represented results [60] because of the volume conduction effect which highly affects the accuracy of the functional connectivity tools [61]. However, DTF has been shown to be insensitive to volume conduction and less sensitive to noise [62]. We will also increase the number of electrodes to improve the accuracy of the functional connectivity analysis in the source space [63,64].

## Conclusion

This study investigated functional alterations in small-world characteristics in patients with benign epilepsy with centrotemporal spikes and showed that the functional organization of BCECTS brain networks changed from an ordered structure in low frequency bands (δ and θ bands) to a less randomly ordered network in higher frequency bands (α band).

This study provides further evidence that the BCECTS brain network is altered. The degree spatial distribution showed that alteration of the functional connectivity in the BCECTS brain was not limited to the epileptogenic zone, but also involved other regions, especially the frontal and occipital regions. The decreased connection density in the occipital and right frontal regions supports functional impairment of these regions. The BCECTS brain with IES, which does not present the features of a small-world network, showed topological characteristics of an ordered network in the δ band and a less ordered network in the α band. A more randomly organized network was also observed in the absence of IES compared to healthy controls.

## Supporting Information

S1 Fig Dipole locations of the averaged spikes for patients. (DOCX)

S2 Fig A sample interictal EEG recording from patient 1. The spikes have been outlined in blue.
(DOCX)

S3 Fig (A) Example of the functional connectivity matrix obtained for Subject 1. (B) The distribution of the PLV values of the functional connectivity matrix; the vertical line shows the optimal threshold. (C) The binarized functional connectivity matrix obtained after applying the optimal threshold.
(DOCX)

S1 Materials (DOCX)
