# Supplementary material for: Functional Brain Dysfunction in Patients with Benign Childhood Epilepsy as Revealed by Graph Theory
Source: PLoS One. 2015 Oct 2;10(10):e0139228. doi: 10.1371/journal.pone.0139228 (PMC4592214; doi:10.1371/journal.pone.0139228)
Supplement: S1 Fig — (DOCX) [file pone.0139228.s001.docx]

S1 Fig. Dipole locations of the averaged spikes for patients.
