# Supplementary figures and images for: Functional Brain Dysfunction in Patients with Benign Childhood Epilepsy as Revealed by Graph Theory
Source: PLoS One. 2015 Oct 2;10(10):e0139228. doi: 10.1371/journal.pone.0139228 (PMC4592214; doi:10.1371/journal.pone.0139228)

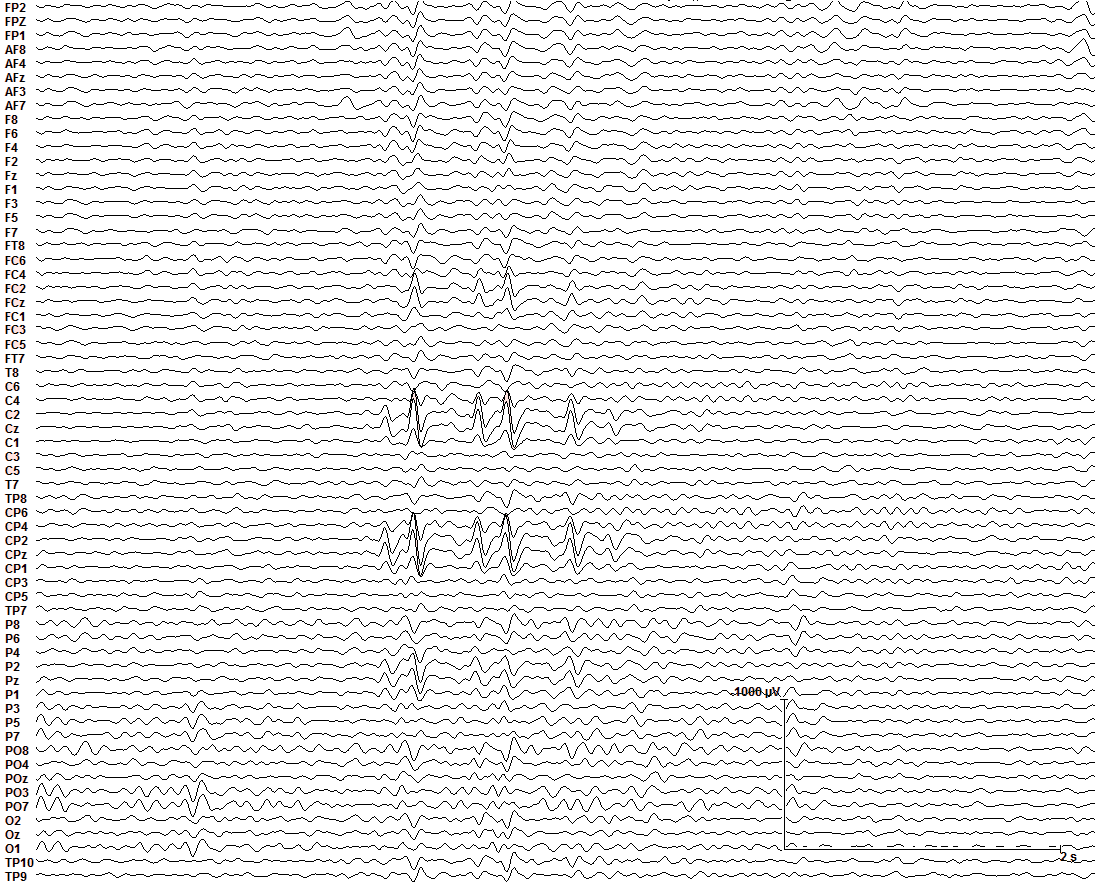


S2 Fig. A sample interictal EEG recording from patient 1. The spikes have been outlined in blue.

Supplement: S2 Fig — The spikes have been outlined in blue. (DOCX) [file pone.0139228.s002.docx]
