# Supplementary material for: Functional Brain Dysfunction in Patients with Benign Childhood Epilepsy as Revealed by Graph Theory
Source: PLoS One. 2015 Oct 2;10(10):e0139228. doi: 10.1371/journal.pone.0139228 (PMC4592214; doi:10.1371/journal.pone.0139228)
Supplement: S3 Fig — (A) Example of the functional connectivity matrix obtained for Subject 1. (B) The distribution of the PLV values of the functional connectivity matrix; the vertical line shows the optimal threshold. (C) The binarized functional connectivity matrix obtained after applying the optimal threshold. (DOCX) [file pone.0139228.s003.docx]

S3 Fig. (a) Example of the functional connectivity matrix obtained for Subject 1, (b) the distribution of the PLV values of the functional connectivity matrix; the vertical line shows the optimal threshold, (c) the binarized functional connectivity matrix obtained after applying the optimal threshold.
