# Supplementary material for: Functional Brain Dysfunction in Patients with Benign Childhood Epilepsy as Revealed by Graph Theory
Source: PLoS One. 2015 Oct 2;10(10):e0139228. doi: 10.1371/journal.pone.0139228 (PMC4592214; doi:10.1371/journal.pone.0139228)
Supplement: S1 Materials — (DOCX) [file pone.0139228.s004.docx]

# **Supporting information**

## **Epileptic subjects**

Dipole source analysis of the centrotemporal spikes region of the patients was performed with Advanced Source Analysis (ASA) software ([http://www.ant-neuro.com](http://www.ant-neuro.com/)). The location of interictal epileptic spikes (IES) was identified for each patient using the spatiotemporal dipole modeling method [1] . All dipoles were oriented in the anterior to posterior direction or *vice versa* and were located in the right precentral and postcentral regions, confirming the homogeneity of the patient population. The C4/C6/CP2/CP6 always recorded IES in BCECTS patients as shown in S2 Fig.

## Functional connectivity

The phase locking value (PLV) was computed from the analytical signals obtained from Hilbert transformation of the band-pass filtered signals, filtered by a linear-phase FIR (finite impulse response) filter. The analytical signals (x (t, f)) were first computed by:

$$\check{x}\left( t,f \right)=x\left( t,f \right)+ix_{H}\left( t,f \right)$$

Where x_H_ (t, f) is the Hilbert transform of the input signals x (t,f).

The phases of the analytical signals were then extracted:

$$\varphi_{x}\left( t,f \right)=\arctan\left( \frac{x_{H}\left( t,f \right)}{x\left( t,f \right)} \right)$$

The relative phase between each channel pair x and y was obtained by:

$$\emptyset\left( t \right)= \varphi_{x}\left( t,f \right)-\varphi_{y}\left( t,f \right)$$

The PLV was finally computed by calculating the exponential of the relative phase:

$$PLV\left( x,y \right)=\frac{1}{N}\left| \sum_{n=1}^{N} e^{i(\emptyset\left( t \right))} \right|$$

Where N is the time point.

# **Computation of graph theory parameters**

## **Degree**

## The degree of a node is the number of links connected to that node. The degree (K) is mathematically defined as:

$$K_{i}=\sum_{J\in N} A_{ij}$$

## Where K_i_ is the degree for the ith node, and A_ij_ is the connection status between nodes i and j. The degree is a characteristic measure of the functional interactions between brain regions.

## **Clustering coefficient**

The clustering coefficient C and characteristic path length L were computed as described in [2,3]. The clustering coefficient C_i_ of the node *i* is defined as:

$$C_{i}=\frac{1}{n}\sum_{i\in N} \frac{2t_{i}}{k_{i}(k_{i}-1)}$$

Where n is the number of nodes, *t_i_* is the number of existing connections among the neighbours of node *i*, and *k_i_* is the actual number of neighbours of node *i* (i.e. degree). The mean clustering coefficient (C) of a network is defined as the mean clustering coefficient over all nodes in the network:

$$C=\frac{1}{N}\sum_{i=1}^{N} C_{i}$$

## **Path length**

The path length L_i_ of a node *i* is defined as

$$L_{i}=\frac{1}{n-1}\sum_{i\neq j} min\{l_{ij}\}$$

Where min{l_ij_} is the shortest absolute distance between the nodes *i* and *j* and the mean path length L over a network is defined as:

$$L=\frac{1}{N}\sum_{i=1}^{N} L_{i}$$

## **Threshold optimization procedure for computation of degree**

Before computing the graph parameters, we applied the threshold (τ) based on the three constraints described in Methods. The first method consists of obtaining one standard deviation above the median connectivity value as shown in S3 Fig. The connectivity matrix before thresholding has shown in Figure A of S3Fig. The distribution of the PLV values has been plotted in Figure B of S3 Fig. In this plot, the vertical pink line represents τ (one standard deviation above the median connectivity value) for a particular subject and frequency band. The optimal threshold was applied to the connectivity matrix (Figure A of S3 Fig) to obtain the binary matrix, as shown in Figure C of S3 Fig.

# **References**

1. Scherg M, Von Cramon D. Two bilateral sources of the late AEP as identified by a spatio-temporal dipole model. Electroencephalogr Clin Neurophysiol Potentials Sect. 1985;62: 32–44. doi:10.1016/0168-5597(85)90033-4

2. Bullmore E, Sporns O. Complex brain networks: graph theoretical analysis of structural and functional systems. Nat Rev Neurosci. 2009;10: 186–198. doi:10.1038/nrn2575

3. Rubinov M, Sporns O. Complex network measures of brain connectivity: uses and interpretations. NeuroImage. 2010;52: 1059–1069. doi:10.1016/j.neuroimage.2009.10.003

# Figure Captions

S1 Fig. Dipole locations of the averaged spikes for patients.

S2 Fig. A sample interictal EEG recording from patient 1*.* The spikes have been outlined in blue.

S3 Fig. (A) Example of the functional connectivity matrix obtained for Subject 1. (B) The distribution of the PLV values of the functional connectivity matrix; the vertical line shows the optimal threshold. (C) The binarized functional connectivity matrix obtained after applying the optimal threshold.
